# Application of Natural Neutrophil Products for Stimulation of Monocyte-Derived Macrophages Obtained before and after Osteochondral or Bone Injury

**DOI:** 10.3390/microorganisms9010124

**Published:** 2021-01-07

**Authors:** Joanna Zdziennicka, Tomasz Szponder, Joanna Wessely-Szponder

**Affiliations:** 1 Department of Pathophysiology, Institute of Preclinical Veterinary Sciences, Faculty of Veterinary Medicine, University of Life Sciences, Akademicka 12, 20-033 Lublin, Poland; joanna.michalska15@gmail.com; 2 Department and Clinic of Animal Surgery, Faculty of Veterinary Medicine, University of Life Sciences, Głęboka 30, 20-612 Lublin, Poland; tomszpon@op.pl

**Keywords:** natural antimicrobial peptides extract, microvesicles, neutrophil degranulation products, monocyte-derived macrophages, osteochondral transplantation, Ti implant, bone graft

## Abstract

We evaluated the use of some neutrophil products, namely; autologous rabbit antimicrobial neutrophil extract (rANE), heterologous porcine antimicrobial neutrophil extract (pANE), neutrophil degranulation products (DGP) and neutrophil microvesicles (MVs) for stimulation of monocyte-derived macrophages (MDMs) to improve healing. Two animal models were evaluated; the rabbit model for autologous osteochondral transplantation (OT) with application of rabbit ANE, DGP or MVs for MDMs stimulation, and the ovine model of the insertion of a Ti implant with the use of porcine ANE, and ovine DGP or MVs for MDMs stimulation. Macrophage activity was assessed on the basis of free radical generation and arginase activity. We estimated that DGP acted in a pro-inflammatory way both on rabbit and ovine MDMs. On the other hand, MVs acted as anti-inflammatory stimulator on MDMs in both experiments. The response to ANE depended on origin of extract (autologous or heterologous). Macrophages from rabbits before and after OT stimulated with autologous extract generated lower amount of NO and superoxide, especially after transplantation. In the ovine model of Ti implant insertion, heterologous ANE evoked increased macrophage pro-inflammatory activity. Our study revealed that these neutrophil products could regulate activity of macrophages, polarizing them into pro-or anti-inflammatory phenotypes that could enhance bone and osteochondral tissue healing.

## 1. Introduction

Inflammation as a response of an organism to local injury involves leukocytes migration to damaged sites to fight bacterial infection and to ensure tissue repair. However, non-resolving inflammation is the cause of many inflammatory disorders such as arthritis and implant rejection [1,2]. Generally, the immune system greatly influences tissue repair in both negative and positive fashion. Therefore, regulation of the immune response during tissue healing seems to be an attractive perspective in regenerative medicine [3].

Neutrophils as important effectors of the innate immunity, are involved in many immune mechanisms. As is well known, they are the first line of defense against bacterial and fungal infections and they release some antimicrobial factors, such as reactive oxygen species (ROS) and proteolytic enzymes, capable of, among other things, eliminating the invading pathogens. Except that, neutrophils are participants of coordination of the general inflammatory response. When neutrophil function is insufficient as in excessive bacterial infection their activity should be enhanced. On the other hand, some disorders are involved in excessive neutrophil activation, in which case attenuation of the neutrophil response is needed [4,5].

There is a continuing debate over the role of neutrophils in bone repair, some authors reported their negative influence on this process. Neutrophils could promote tissue injury by release of some enzymes such as elastase, and free radicals generation and, furthermore, the neutrophil-induced inflammatory response could exacerbate the already existing tissue damage [6]. Other authors found that neutrophil depletion in a rat model of growth plate injury promoted osteogenic but suppressed chondrogenic differentiation of progenitor cells [6,7]. Bone repair was also impaired due to increased ROS generation in a rat model, thus a balanced neutrophil activation appeared important for normal fracture healing [6]. Many studies are aiming to manipulate neutrophils for enhance healing, including targeting neutrophil maturation, interfering with their accumulation at the site of injury and reversing the detrimental changes of neutrophil function. Neutrophil granule-derived products appeared to have several roles, from directly killing microorganisms to activating other inflammatory cells, such as monocytes or macrophages [8]. Activated macrophages are able to differentiate into two distinct subpopulations. The initial inflammatory response is carried out by classically activated pro-inflammatory M1 macrophages, with potential for killing of invading pathogens. Then, the resolution phase is carried out by M2 macrophages involved in immune suppression and tissue remodeling. Thus, macrophages can adopt a variety of functional phenotypes depending on changes in the tissue microenvironment. Different stimuli can influence the macrophages polarization, with potentially dangerous consequences if not appropriately regulated. Classically activated M1 macrophages can cause tissue injury, whereas normally pro-resolving M2 macrophages can promote fibrosis or exacerbate allergic responses in case of abnormal activity [3,5,9]. As estimated previously, some secretion products derived from neutrophils are able to modulate macrophage function and in this way the effectiveness of the immune response in host defense [10].

Antimicrobial peptides (AMP) apart from their direct microbicidal role are known as compounds with immunomodulatory function. Some of them, e.g., human cathelicidin LL-37 suppress pro-inflammatory activation of macrophages, and promote angiogenesis and wound healing [11]. Previous studies indicated that neutrophil AMP extracts are the mixtures containing different peptides with confirmed antibacterial activity; cathelicidins and defensins in rabbits or only cathelicidins in pigs [12]. To date some neutrophil-derived products were considered as means for controlling the immune system to promote tissue repair. Among them, autologous and heterologous AMP extracts and neutrophil degranulation products (DGP) were used for regulation of the macrophage function [13,14,15,16,17]. Moreover, since neutrophil-derived microvesicles (MVs) exert anti-inflammatory and pro-resolving properties [18], we evaluated the influence of these structures on macrophage function during repair process.

Implanted biomaterials can significantly influence the immune response and macrophage polarization, either enhancing or reducing inflammation depending on their physicochemical properties [3]. Titanium (Ti) is the most common biomaterial used for orthopedic implants. However, Ti implants may cause excessive immune response leading to high implantation failure rates. This is caused by aggregates of Ti ions with serum proteins, which activate and stimulate release of the cytokines from human macrophages [19,20].

Knowledge about the mechanisms involved in degeneration of the cartilage surrounding osteochondral defects which leads to progression of osteoarthritis (OA) is still incomplete [21]. Macrophages play a crucial role in this condition because of their phenotype, that may range from pro-inflammatory to anti-inflammatory. Pro-inflammatory macrophages exacerbate the progression of cartilage degeneration whereas anti-inflammatory macrophages can inhibit this process. Thus, modulation by directing suppression of pro-inflammatory macrophages or stimulation of anti-inflammatory macrophages should be considered in developing new therapies aiming at inhibiting cartilage degeneration causing OA [22].

To assess the potential of neutrophil-derived products for modifying of the macrophage response we prepared two animal models; the rabbit model of autologous osteochondral graft and the ovine model of insertion of Ti implant into the tibial growth plate. Two different orthopaedic models and two different surgical procedures were considered for assessment of repair process in two different tissues of musculoskeletal system. In the rabbit model we assessed the in vitro response of rabbit monocyte-derived macrophages (MDMs) to autologous rabbit antimicrobial neutrophil extract (rANE), rabbit neutrophil degranulation products (DGP) or rabbit neutrophil microvesicles (MVs). In the ovine model, MDMs were treated in vitro with heterologous porcine neutrophil extract (pANE), ovine DGP or ovine neutrophil MVs.

## 2. Materials and Methods

### 2.1. Rabbit Model of Osteochondral Defect

#### 2.1.1. Animals and Surgical Procedures

For the experiment, eight New Zealand white rabbits, male, 6 months old, weighing 3.5–4.0 kg were used. The experiment was conducted in accordance with the International Council for Laboratory Animal Science Guidelines for the care and the use of laboratory animals and was approved by the Local Ethics Committee Number II in Lublin (52/2017).

**Figure 1:**
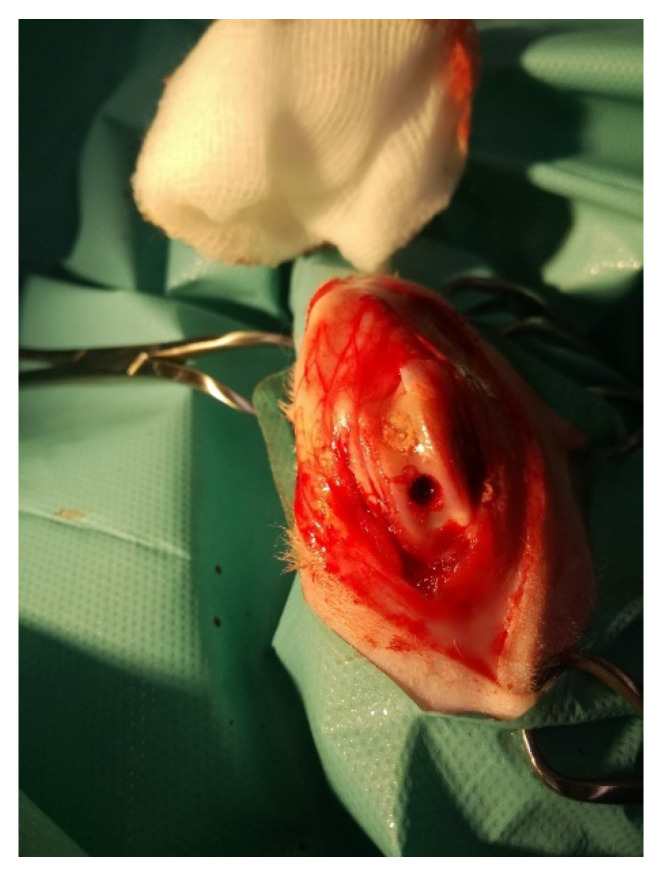
The rabbit stifle joint with a cartilage defects in the course of osteochondral autograft transplantation.

For anaesthesia, isofluorane (Iso-Vet^TM^, Piramal Healthcare Ltd., West Drayton, UK) was used. The autologous osteochondral transplantation (OT) was performed with the use of specially designed instrumentation (Smith and Nephew Inc., Andover, MA, USA). The procedure included the collection of full thickness grafts and transplantation from one knee joint into the other in the same rabbit (Figure 1).

#### 2.1.2. Preparation of Blood-Derived Products

In the course of the experiment two different neutrophil granule products, namely ANE and DGP were used for the study. The content of these products was evaluated in previous studies performed by our group and other authors. As estimated previously [13] ANE contains short cationic antimicrobial peptides, cathelicidins, defensins, and their fragments. DGP was the product of degranulation and consisted of degranulation products of stimulated neutrophils [17]. Both products were prepared simultaneously and frozen for further use.

##### Preparation of Rabbit Antimicrobial Neutrophil Extract (rANE)

Blood was drawn from the marginal auricular vein at two time points; 7 days before OT and 2 h after OT. For the preparation of rANE, blood from the first time point of sampling was used. After treatment with 0.83% ammonium chloride for 10 min the blood was centrifuged at 700× *g* for 15 min at 4 °C. The cell count and viability were assessed using an R1 Automated Cell Counter (Olympus, Warsaw, Poland) then isolated neutrophil suspension was homogenized and released neutrophil granules were collected by centrifugation at 25,000× *g*, 40 min, 4 °C and stirred overnight in 10% acetic acid at 4 °C. The extract containing the antimicrobial peptides (ANE) was separated from the granules (25,000× *g*, 20 min, 4 °C), lyophilized and stored at −20 °C. The lyophilized portions were resuspended in the culture medium to a concentration of 20 μg/mL immediately before further experiments [23].

##### Neutrophil Degranulation Products (DGP) and Their Activity

Blood samples from the rabbits were taken 7 days before OT and used for isolation of neutrophils according the method described above. DGP was the products of degranulation of isolated neutrophils (density 1.0 × 10^6^ cells/mL), induced by *N*-formylmethionyl-leucyl-phenylalanine (fMLP, 1 μg/mL, Sigma-Aldrich, Poznan, Poland). After 10 min of incubation at 37 °C neutrophils were centrifuged (300× *g*, 10 min) and cell-free supernatant containing DGP was collected and stored at −20 °C until later use [17]. Protein concentration was determined by Lowry’s method using bovine serum albumin BSA as a standard.

Analysis of the activity of neutrophils degranulation product was conducted on the basis of activity of two enzymes released from azurophilic granules, namely elastase and myelopreoxidase (MPO), and one enzyme from specific granules, namely alkaline phosphatase (ALP) [13]. Elastase activity was assessed spectrophotometrically on the basis of cleavage of a substrate-azocasein (Sigma-Aldrich). The equal volume of cell suspension was incubated with the substrate at 25 °C for 10 min; and then absorbance was measured at 490 nm using a BioTek EL800 microplate reader (BioTek, Janki, Poland). MPO release was estimated after 10 min incubation of the sample with the equal volume of o-phenylendiamine (Sigma-Aldrich, Poznan, Poland) as a substrate. ALP release was determined after incubation in the same conditions (10 min, 25 °C) with the equal volume of 4-nitrophenyl phosphate disodium salt hexahydrate (Sigma-Aldrich, Poznan, Poland), then, absorbance was measured at 405 nm. Results obtained for each enzyme were compared to maximal neutrophil degranulation (100% release) after treatment with 0.5% Triton X-100 (Sigma-Aldrich, Poznan, Poland) and expressed as % of maximal enzyme release.

Nitric oxide (NO) generation was determined on the basis of nitrite measurement as the stable product of NO in culture medium. Briefly, 100 µL of the culture supernatant and 100 µL of Griess reagent (0.1% *N*-[1-naphtyl] ethylendiamine dihydrochloride 1% sulphanilamide and 2.5% H_3_PO_4_) were mixed and incubated at room temperature for 10 min and then absorbance was measured. Values obtained were converted into micromoles (µM) of NaNO_2_ based on a standard curve (1.25, 2.5, 5, 10, 20, 40, 80 µM of Na_2_NO_2_). Superoxide anion generation was measured colorimetrically (absorbance read at 545 nm) after incubating neutrophils with 0.1 mg/mL nitroblue tetrazolium (NBT, Sigma-Aldrich, Poznan, Poland) solution in phosphate-buffered saline (PBS) at room temperature for 10 min [14].

##### Preparation of Microvesicles (MVs)

The suspension of isolated neutrophils (95% viable, >75% purity in May–Grunwald–Giemsa-stained preparations, density 2 × 10^7^) was incubated with 1 μg/mL fMLP for 20 min at 37 °C 5% CO_2_. After incubation neutrophils were centrifuged (4400× *g* 15 min, 4 °C). Obtained supernatant was centrifuged again 20,000× *g* 30 min, 4 °C and pellet was resuspended in PBS at the original incubation volume. Protein concentration was determined by the Lowry’s method using BSA as standard [18].

#### 2.1.3. Monocyte-Derived Macrophages (MDMs) Culture and Activity

Peripheral blood mononuclear cells (PBMCs) were isolated as described previously [14] and cultured at a density of 1.0 × 10^6^ cells/mL into 96-well flat-bottomed tissue culture plates (Nunc, ThermoFisher Scientific, Warsaw, Poland), at 37 °C and 5% CO_2_ for 24 h in Dulbecco’s Modified Eagle’s Medium (DMEM) with 10% bovine calf serum (BCS, Biomed, Lublin, Poland). Cultures were microscopically evaluated (Olympus CK-40) and when cells differentiated into macrophages we started the experiment. Then, the cultures without additional stimulation were control groups (BCS), whereas other were stimulated with DGP, neutrophil MVs or ANE. Measurements of MDMs activity were conducted after 24 and 72 h of incubation at 37 °C with 5% CO_2_. The functional analysis of MDMs cultures after different stimulation was conducted on the basis superoxide and NO generation and assessment of arginase activity in vitro, as described previously [13,24].

### 2.2. Sheep Model of Insertion of Ti Implant into the Tibial Growth Plate

#### 2.2.1. Animals and Surgical Procedures

**Figure 2:**
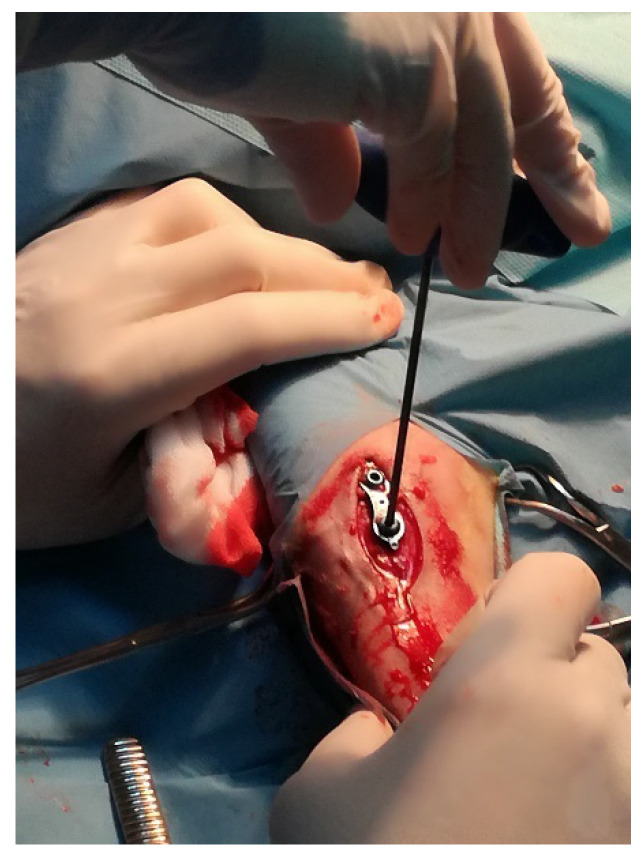
The implantation of titanium plate into the tibial growth plate in sheep.

The study was performed in a group of 8 sheep, females, BCP local breed, 4 months old, about 20 kg body weight, derived from the Bezek Experimental Farm, University of Life Sciences, Lublin. All animals were fed, housed and cared according to the rules concerning the protection of animals. The experiment was approved by the local Ethics Committee No II in Lublin (No 84/2019). For the surgery, animals were anaesthetized with xylazine (0.1 mg/kg intramuscularly) and butorphanol (0.1 mg/kg). Additionally, for local anaesthesia 2% lignocaine was used. The proximal tibia was exposed with standard surgical approach. Then a titanium plate was implanted directly into the proximal growth plate of the tibia (Figure 2).

#### 2.2.2. Preparation of Blood-Derived Products

##### Blood Sampling, Cell Cultures and Blood-Derived Products

Porcine ANE was prepared as previously described [25]. The solution containing the peptides was separated from the granules, lyophilized and stored at −70 °C. Blood samples for neutrophil in vitro assays were collected into EDTA tubes and the cells were plated at a density of 1.0 × 10^6^ cells/mL. DGP preparation was conducted similarly as in rabbit model and cell-free supernatant was collected and stored at −20 °C until later [17]. Cultures were incubated for 30 min at 37 °C in the presence of 5% CO_2_. Enzyme release, NO level and superoxide anion generation were measured as in a previously described rabbit model.

#### 2.2.3. MDMs Culture and Activity

Isolation of blood derived monocytes, culture and functional assessment were conducted similarly as for preparation and evaluation of rabbit MDMs. Blood for preparation of neutrophil-derived products was collected 7 days before insertion of the Ti implant. At that time hematological tests were also conducted. Monocytes for MDMs cultures were isolated 7 days before Ti implant insertion and 2 h after implantation. These cells were stimulated with DGP, neutrophil MVs or pANE or left without additional stimulation and marked as BCS MDMs (control). Functional analyses of MDMs were conducted in two measurements; after 24 h and 72 h of incubation at 37 °C with 5% CO_2_ as described in the rabbit model.

### 2.3. Statistical Analysis

All in vitro experiments were performed at least three times, and the quantitative data were expressed as mean ± standard deviation. Data obtained were analyzed with Statistica 13.1 (StatSoft, Cracow, Poland). Significance was calculated using a one-way analysis of variance (ANOVA) followed by a Tukey’s test and *p* < 0.05 was set as statistical significance.

## 3. Results

### 3.1. Rabbit Model of Osteochondral Defect

#### 3.1.1. Neutrophils Secretory Activity

Isolated neutrophils were used for preparation of neutrophil-derived autologous products. Activity of neutrophils culture used for preparation of DGP was assessed on the basis of enzyme release and NO or superoxide generation and results were summarized in Table 1. The enzyme activity was compared to maximal release in neutrophil cultures treated with 0.5% Triton X-100 (Sigma-Aldrich) and expressed as percent of activity.

#### 3.1.2. Rabbit MDMs Response to Neutrophil-Derived Products

**Figure 3:**
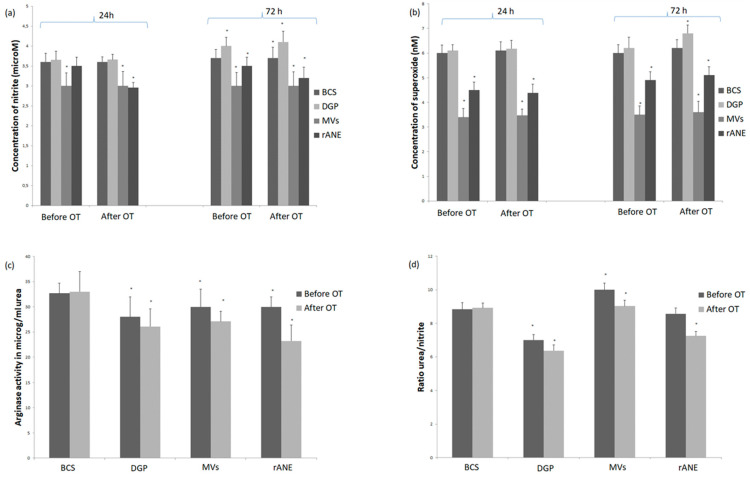
Generation of (**a**) nitric oxide, (**b**) superoxide and (**c**) arginase activity with (**d**) ratio urea/nitrite in monocyte-derived macrophages (MDMs) from rabbits (*n* = 8) before osteochondral transplantation (OT) and 2 h after OT. Cultures stimulated only with Dulbecco’s Modified Eagle’s Medium (DMEM) with 10% bovine calf serum (BCS) and stimulated with neutrophil degranulation products (DGP), or neutrophil-derived microvesicles (MVs) or rabbit antimicrobial neutrophil extract (rANE) for 24 h and for 72 h shown in the Figure 3a,b. Data present mean values ± standard error (SE) of at least three replicates for each bar at both time points. * *p* < 0.05 values differ significantly compared with BCS after 24 h incubation (**a**,**b**). Arginase activity (**c**) and ratio urea/nitrite (**d**) in lysates of MDMs cultures stimulated with BCS, DGP, MVs or rANE. Data present mean values ± SE of at least three replicates for each bar. * *p* < 0.05 values differ significantly compared with BCS (**c**,**d**).

The experiment was conducted on MDMs cultured from monocytes isolated from rabbits blood seven days before and 2 h after OT. After stimulation with DGP macrophages obtained in both time-points showed pro-inflammatory activity assessed on the basis of NO and superoxide generation. These cultures generated higher amounts of NO in comparison with BCS ones. Moreover, significant difference (*p* < 0.05) in measurement after 72 h incubation in comparison with results in measurement after 24 h incubation was observed (Figure 3a). Superoxide generation was also significantly (*p* < 0.05) higher in cultures stimulated with DGP in measurement after 72 h in group after OT (Figure 3b). On the other hand, arginase activity was significantly lower (*p* < 0.05) in both cultures before and after OT compared to the BCS groups (Figure 3c). Similar decrease was noted in ratio urea/nitrite. Obtained values in studied groups (both before and after OT) in rabbits indicated pro-inflammatory response of cultures of macrophages stimulated with DGP (Figure 3d).

Addition of MVs-enriched pellet into the macrophage cultures induced significant decrease of NO generation at both time points: before and after transplantation and in both measurements after 24 and 72 h of incubation (Figure 3a). Similarly, superoxide generation was significantly lower compared to BCS group and at both time-points (before and after OT) and in both measurements (24 and 72 h incubation) (Figure 3b). Arginase activity significantly decreased in MDMs group treated with MVs-enriched pellet in comparison with BCS group, whereas the ratio urea/nitrite, was significantly elevated in both MVs groups before and after OT (Figure 3c,d).

After addition of rANE, decrease of NO generation by MDMs was observed in all groups. This decrease was slight and insignificant in groups before OT after 24 h incubation and more pronounced in groups after OT (Figure 3a). Addition of rANE into cultures significantly diminished (*p* < 0.05) superoxide production in all studied groups and measurements (Figure 3b). Supplementation with rANE evoked decrease of arginase activity from value 32.7 ± 2.0 μg/mL urea in the MDMs BCS group before OT and 33.00 ± 1.1 μg/mL urea in MDMs BCS after OT to values 30.0 ± 2.33 μg/mL urea and 23.21 ± 2.11 μg/mL urea, respectively, in groups treated with rANE (Figure 3c). Similarly ratio urea/nitrite was significantly decreased in MDMs group after implantation compared to both groups without stimulation and group before OT (Figure 3d).

### 3.2. Insertion of Ti Implant in the Ovine Model

#### 3.2.1. Neutrophils Secretory Activity

Isolated ovine neutrophils were used for preparation of neutrophil-derived autologous products, namely DGP and MVs. Results of measured activity of neutrophils culture used for preparation of DGP were summarized in Table 2. The enzyme activity was compared to maximal release in neutrophil cultures treated with 0.5% Triton X-100 (Sigma-Aldrich) and expressed as percent of activity.

#### 3.2.2. MDMs Response to Neutrophil-Derived Products

**Figure 4:**
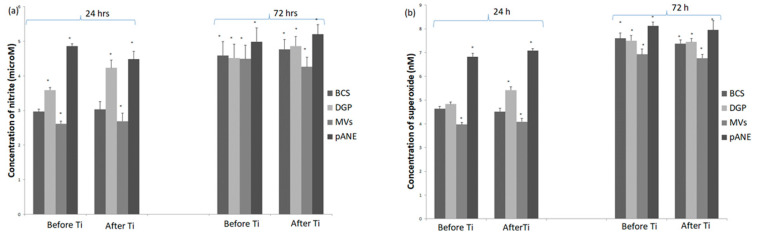

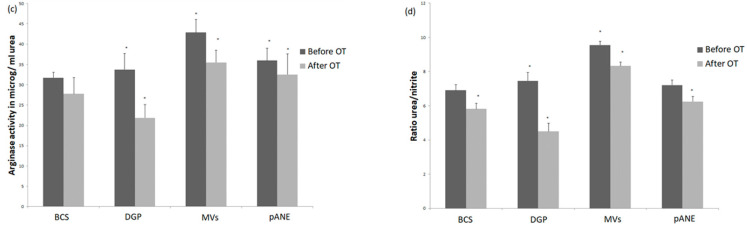
Generation of (**a**) nitric oxide, (**b**) superoxide and (**c**) arginase activity with (**d**) ratio urea/nitrite in MDMs from sheep (*n* = 8) 7 days before and 2 h after Ti implant insertion into the tibia. Cultures stimulated only with DMEM with 10% BCS and stimulated with neutrophil degranulation products (DGP), or neutrophil-derived microvesicles (MVs) or porcine antimicrobial neutrophil extract (pANE) for 24 h and for 72 h shown in the Figure 4a,b. Data present mean values ± SE of at least three replicates for each bar at both time points. * *p* < 0.05 values differ significantly compared with BCS after 24 h incubation (**a**,**b**). Arginase activity (**c**) and ratio urea/nitrite (**d**) in lysates of MDMs cultures stimulated with BCS, DGP, MVs or pANE. Data present mean values ± SE of at least three replicates for each bar. * *p* < 0.05 values differ significantly compared with BCS (**c**,**d**).

We estimated that after stimulation with DGP MDMs cultures generated significantly higher amounts of NO in comparison with BSC MDMs groups in measurement after 24 h incubation at 37 °C and 5% CO_2_, whereas in the second measurement (72 h incubation) the differences were insignificant (Figure 4a). In cultures obtained after Ti implant insertion generation of superoxide after 24 h incubation with DGP was elevated to the value 5.42 ± 0.15 nM compared to values obtained in the BCS group (4.52 ± 0.18 nM). In cultures of MDMs obtained before implantation differences before the BCS group and DGP group were insignificant in both measurements (24 h and 72 h incubation) (Figure 4b). Arginase activity after the addition of DGP was significantly diminished in MDMs cultures after implantation, compared to values before implantation and to both BCS groups before and after implantation (*p* < 0.05) (Figure 4c). The ratio urea/nitrite reflected changes in arginase activity (Figure 4d).

Addition of MV-enriched pellet into the macrophage cultures induced decrease of NO generation before and after implantation after 24 h of incubation, whereas after 72 h incubation, a significant decrease of NO generation was noted only in the group after implantation (Figure 4a). Stimulation of MDMs with neutrophil MVs also decreased superoxide production which was noted in all studied cultures (Figure 4b). Arginase activity, in turn, was significantly higher in comparison with the BCS group in both cultures before and after implantation (Figure 4c). A similar effect was observed in results of urea/nitrite ratio of the studied MDMs cultures (Figure 4d). Addition of porcine pANE acted in a pro-inflammatory way and caused a significant increase of NO generation in both groups (before and after implantation) and both measurements (after 24 and 72 h of incubation). A similar effect was noted in the case of superoxide generation (Figure 4a,b). Arginase activity was elevated in comparison with group stimulated only with BCS, whereas changes in ratio urea/nitrite were insignificant (Figure 4c,d).

## 4. Discussion

Our study revealed that autologous or heterologous neutrophil-derived products could modulate the activity of macrophages, polarizing them into pro- or anti-inflammatory phenotypes. This response may be crucial for repair process in osteochondral tissues or bone regeneration. Previous reports underlined that neutrophils are not only directly microbicidal cells but are also regulators of macrophage function [10,15,16,26]. To extend and deepen previous research we evaluated the response of MDMs to neutrophil-derived products, namely; DGP, neutrophil MVs and autologous or heterologous antimicrobial extracts on two animal models, rabbit and ovine, of osteochondral and bone repair, respectively.

First we evaluated in vitro activity of degranulation products of isolated rabbit and ovine neutrophils. Obtained results indicated that stimulated neutrophils released elastase, MPO from primary granules, ALP from secondary granules, and generated superoxide and NO. As estimated previously proteins from degranulated neutrophils stimulated monocytes and may alter macrophage function [10,15,16,17,26]. They trigger an active response involved in enhanced bacterial phagocytosis and reactive oxygen species (ROS) formation [16]. Additionally, neutrophil-derived elastase activated macrophages via TLR-4 inducing production and release of TNF. Thus, neutrophil secretion products modulate microbicidal mechanisms in monocytes and macrophages, for enhancing of the effectiveness of innate immunity [10].

We estimated that in both our models DGP acted as pro-inflammatory stimulator of macrophages. This study revealed that both rabbit and ovine DGP enhanced NO and superoxide production by MDMs in vitro. This response in the rabbit model was noted in both groups before and after osteochondral transplantation after 72 h MDMs incubation at 37 °C and 5% CO_2_, whereas the response in the ovine model was more pronounced after 24 h of incubation in both MDMs cultures before and after implantation of the Ti plate into the tibia. According to Hussen et al. bovine DGP do not polarize monocyte–derived macrophages but differentiated them towards a mixed phenotype with enhanced antimicrobial functions [17]. In our experiment, antimicrobial functions were not evaluated, but pro-inflammatory response was confirmed on the basis of increased NO, and superoxide generation, and decreased arginase activity. These markers were previously used for functional differentiation of macrophage subsets [9,27,28].

In the next step of the experiment we estimated that after stimulation with DGP arginase activity decreased in both rabbit MDMs cultures before and after OT in comparison with BCS stimulated macrophages. In the ovine model of Ti implant insertion, decrease of arginase activity in response to DGP was noted only in the group of MDMs obtained after implantation. These results were consistent with the research of Campbell et al. [29] which indicated that arginase activity is dynamically regulated during healing and its increase is associated with M2 alternatively activated macrophages not pro-inflammatory M1 subsets. The balance between inducible NO synthase (iNOS) and arginase activity is tightly regulated during the repair process and arginase activity is strongly involved in this process. Therefore, the absence of iNOS delayed healing, whereas upregulation of iNOS activity correlated with faster healing and local macrophage polarization significantly influences the tissue repair process [29].

For better functional analysis of interactions between NO generation and arginase activity in MDMs cultures we calculated urea/nitrite ratio, which was previously introduced by Geelhaar-Karsch et al. [27]. In our experiment on the rabbit model the ratio urea/nitrite in MDMs cultures stimulated with DGP was lower than in BCS MDMs and this effect was noted at both time points before and after OT. Conversely, in the ovine model only the response after implantation was noticeably lower compared to BCS MDMs.

Another stimulator used in our study was neutrophil MVs-enriched pellet. Extracellular microvesicles are cell-derived membrane fragments of granules generated by most eukaryotic cells. These structures can transfer biologically active molecules participating in cell adhesion, chemotaxis and angiogenesis [30]. In the study of Dali and Serhan, MVs obtained from activated neutrophils were used for stimulation of macrophages for pro-resolving response [1]. Similarly, in the work of Gasser and Schifferli [31] neutrophil-derived MVs down-regulated macrophage inflammatory response. In the rabbit model we observed a significant decrease of NO and superoxide generation by MDMs at both time points after treatment with MVs-enriched pellets. This response confirmed anti-inflammatory function of MVs. Also in the ovine model similar effect on MDMs function was noted both before and after implantation of biomaterial. Previous study has shown that neutrophil-derived MVs exert anti-inflammatory responses by suppressing the activation of immune cells such as dendritic cells, monocytes, and macrophages. Since MVs are generated from neutrophils in tissues, they are presumably involved in limiting excessive inflammation by suppression of surrounding inflammatory cells [32].

In the rabbit model we observed decreased arginase activity in MDMs cultures obtained before and after OT and stimulated with MVs-enriched pellets in comparison with BCS MDMs. On the other hand, in our ovine model, increase of arginase activity in both MDMs cultures before and after implantation was observed. Rhys et al. estimated that MVs alone did not affect macrophage phenotype, suggesting that they inhibit classical activation rather than imparting alternative activation. According to these authors, potential therapeutic application of neutrophil MVs could be considered during treatment of arthritis [18].

Depending on the specific biochemical conditions, the NO/L-ornithine imbalance may be protective or harmful to the target cells or tissues; thus, understanding of the main regulatory mechanisms of the arginase/NOS interplay is crucial for the development of successful diagnosis and treatment [33]. In the rabbit model we observed the decrease of arginase activity, together with the increased ratio arginase/nitrite. In the study of Shahbazi et al. this ratio increased in cultures of macrophages after anti-inflammatory stimulation for 24 and 48 h [34]. Previously it was estimated that M0 macrophages treated with neutrophil-derived MVs showed the increased expression of anti-inflammatory markers such as Arg-1, which confirmed the differentiation of M0 macrophages into M2 subsets. This polarization is important for tissue remodeling and immunoregulation [32]. In our ovine model the anti-inflammatory response of M2 macrophages was involved in the enhanced amount of urea and elevated ratio of urea/nitrite compared to control MDMs cultures. Geelhaar-Karsch noted a similar effect in M2 macrophages from chronic multisystemic infection (CWD) patients in comparison with healthy donors [27].

In our experiment, macrophage response to antimicrobial extract depended on the origin of extract (rabbit or porcine ANE), animal species (rabbit or sheep) and the status of animal models (before or after surgical procedure). We estimated that macrophages from rabbits before and after osteochondral transplantation stimulated with autologous extract generated a lower amount of NO, especially after transplantation both in cultures incubated for 24 and 72 h. Superoxide generation and arginase activity were also diminished in all studied groups of MDMs. The results obtained were consistent with our previous findings [23]. In the ovine model of Ti implant insertion, heterogenous (porcine) antimicrobial extract evoked increased macrophage activity in respect of NO and superoxide generation, also arginase activity was elevated. These results indicated that this extract enhanced pro-inflammatory functions related to NO and superoxide generation, without diminishing of arginase activity which is crucial for the repair process. Soehnlein et al. stated that neutrophil secretions containing antimicrobial peptides murine and human origin are mediators of the macrophage response to neutrophils and these secretions are involved in regulation of the immune resistance to bacterial infections by direct activation of macrophages [15]. Some authors found that human cathelicidin LL-37 specifically stimulated mobilization of inflammatory mice monocytes [10].

## 5. Conclusions

We revealed that some neutrophil-derived products, namely; autologous ANE and MVs can cause an anti-inflammatory response of macrophages, whereas others, such as DGP or heterologous ANE, can cause pro-inflammatory activity of these cells. The macrophage responses to neutrophil-derived products during two different tissue repair processes were similar, thus the results obtained may be applicable in different species and different repair process models. These results gives rise to consideration of the use of some natural neutrophil-derived products in different disorders of the musculoskeletal system, however, further research is needed. The importance of this finding lies in the potential of developing innovative therapeutic approaches based on autologous and heterologous neutrophil-derived products for modulation of the macrophage response. To summarize, precise and time-optimized regulation of inflammation is crucial to normal bone osteochondral tissue repair. Understanding of the complex and often contradictory role of inflammation in the musculoskeletal system is needed to develop novel and effective therapies for a variety of pathological conditions. In this context, modulation of macrophage polarization that enhances bone and osteochondral tissue healing represents a new potential strategy for treatment.

## Figures and Tables

**Table 1:** Activity of rabbit neutrophils isolated before and after osteochondral transplantation (OT) assessed on the basis of enzyme release and free radical generation.

Neutrophil Enzyme/Reactive Oxygen Species (ROS)	Control-Cultures without *N*-formylmethionyl-leucyl-phenylalanine (fMLP) Stimulation	Before Implantation	After Implantation
Elastase (% activity)	49.70 ± 0.5	50.00 ± 0.8	51.00 ± 0.69
Myeloperoxidase (MPO % activity)	23.03 ± 0.5	23.45 ± 0.49	32.51 ± 0.57
Alkaline phosphatase (ALP % activity)	22.00 ± 0.1	22.89 ± 2.8	22.59 ± 2.4
Nitric oxide (NOµM of nitrite)	2.5 ± 0.5	2.5 ± 0.4	3.0 ± 0.5
Superoxide (nM)	3.0 ± 0.25	3.19 ± 0.3	3.14 ± 0.2

**Table 2:** Activity of ovine neutrophils isolated before and after Ti implant insertion assessed on the basis of enzyme release and free radical generation.

Neutrophil Enzyme/ROS	Control (Cultures without fMLP Stimulation)	Before Implantation	After Implantation
Elastase (% activity)	50.72 ± 0.53	50.3 ± 3.88	54.2 ± 2.18
MPO (% activity)	24.13 ± 0.2	24.9 ± 2.3	23.3 ± 0.49
ALP (% activity)	14.80 ± 0.2	15.44 ± 2.31	14.84 ± 1.32
NO (µM of nitrite)	2.8 ± 0.5	3.0 ± 0.1	3.0 ± 0.02
Superoxide (nM)	4.5 ± 0.3	5.5 ± 0.11	4.8 ± 0.1

## Data Availability

Data is contained within the article.
